# News from the Cold Chamber: Clinical Experiences of POLARx versus Arctic Front Advance for Single-Shot Pulmonary Vein Isolation

**DOI:** 10.3390/jcdd9010016

**Published:** 2022-01-08

**Authors:** Denise Guckel, Philipp Lucas, Khuraman Isgandarova, Mustapha El Hamriti, Leonard Bergau, Thomas Fink, Vanessa Sciacca, Guram Imnadze, Martin Braun, Moneeb Khalaph, Georg Nölker, Philipp Sommer, Christian Sohns

**Affiliations:** 1 Clinic for Electrophysiology, Herz-und Diabeteszentrum NRW, Ruhr-Universität Bochum, 32545 Bad Oeynhausen, Germany; dguckel@hdz-nrw.de (D.G.); plucas@hdz-nrw.de (P.L.); kisgandarova@hdz-nrw.de (K.I.); melhamriti@hdz-nrw.de (M.E.H.); lbergau@hdz-nrw.de (L.B.); tfink@hdz-nrw.de (T.F.); vsciacca@hdz-nrw.de (V.S.); gimnadze@hdz-nrw.de (G.I.); mbraun@hdz-nrw.de (M.B.); mkhalaph@hdz-nrw.de (M.K.); g.noelker@hospitalverbund.de (G.N.); csohns@hdz-nrw.de (C.S.); 2 Clinic for Internal Medicine II/Cardiology, Christliches Klinikum Unna Mitte, 59423 Unna, Germany

**Keywords:** atrial fibrillation, catheter ablation, cryoballoon, single-shot ablation devices

## Abstract

Cryoballoon (CB)-guided pulmonary vein isolation (PVI) represents a cornerstone in the treatment of atrial fibrillation (AF). Recently, a novel balloon-guided single shot device (POLARx, Boston Scientific) was designed. Our study aimed to compare the efficacy, safety and characteristics of the novel CB system with the established one (Arctic Front Advance (Pro), AFA, Medtronic). A total number of 596 patients undergoing CB-guided ablation for AF were included. 65 patients (65.0 ± 11.6, 31% female) undergoing PVI with the POLARx were compared to a cohort of 531 consecutive patients (63.0 ± 27.9, 25% female) treated with AFA. Acute PVI was achieved in all patients (n = 596, 100%). Total procedure duration (POLARx 113.3 ± 23.2 min, AFA 100.9 ± 21.3 min; *p* < 0.001) and fluoroscopy time (POLARx 10.5 ± 5.9 min, AFA 4.8 ± 3.6 min; *p* < 0.001) were significantly longer in the POLARx group. The POLARx balloon achieved significantly lower nadir temperatures (POLARx −57.7 ± 0.9 °C, AFA −45.1 ± 2.6 °C; *p* < 0.001) and a significantly higher percentage of pulmonary veins successfully isolated with the first freeze (*p* = 0.027 *). One major complication occurred in the POLARx (2%) and three (1%) in the AFA group. Both ablation systems are comparably safe and effective. AF ablation utilizing the POLARx system is associated with longer procedure and fluoroscopy times as well as lower nadir temperatures.

## 1. Introduction

Cryoballoon (CB)-guided catheter ablation is an effective treatment for atrial fibrillation (AF) [1,2,3]. Complete pulmonary vein isolation (PVI) represents the cornerstone of this procedure [1]. As CB-guided single-shot PVI is associated with shorter procedure times, an improved learning curve and a higher degree of lesion reproducibility it provides a valid alternative to radiofrequency (RF)-guided catheter ablation [1,4,5,6,7]. Over the past 15 years, much experience has been gained with the established AFA-CB (Arctic Front Advance (Pro), AFA, Medtronic) [1,5,8,9,10,11,12,13].

Meanwhile, a second CB-system (POLARx, Boston Scientific) became available. First clinical experiences with this novel system reported comparable efficacy and safety, but differences in terms of biophysiological parameters [14,15,16,17,18,19,20,21,22,23].

This study aimed to access clinical performance in terms of lesion formation and procedural safety of the novel balloon device in comparison to the established CB-system for AF ablation under routine clinical conditions.

## 2. Methods

This observational study included 596 consecutive patients undergoing CB-guided catheter ablation for symptomatic and drug refractory paroxysmal (PAF) and persistent (PERS) AF between January 2013 and August 2021. We compared clinical characteristics and acute procedural outcomes of 65 patients undergoing single-shot device-guided PVI utilizing the POLARx versus another cohort of 531 patients treated with AFA. All procedures were performed by experienced electrophysiologists of our hospital according to institutional standards.

### 2.1. Procedural Management

LA/LAA thrombus formation was ruled out in all patients prior to the ablation procedure. Preprocedural imaging (MRI or CT) was performed in all patients for procedural planning and to evaluate the individual anatomical considerations of the LA and PVs. AADs except for amiodarone were discontinued at least three half-lives before ablation. Anticoagulation with phenprocoumon was continued aiming for an International Normalized Ratio (INR) between 2.0 and 3.0. Direct oral anticoagulants (DOAC) were stopped one half-life before ablation. Pericardial effusion (PE) was ruled out immediately after ablation and the next day 4 h thereafter. Anticoagulation was continued within 4 h after the procedure with phenprocoumon or DOAC when there was no evidence for PE. Phrenic nerve palsy was assessed as transient if it completely resolved during the inpatient stay. Phrenic nerve palsy persisting beyond this was declared persistent. AADs were prescribed to the operators’ discretion for a period of 3 months following ablation.

### 2.2. Ablation Procedure

The procedure was performed under conscious sedation with propofol and analgesia with fentanyl as required.The 28-mm AFA cryoballoon (Arctic Front Advance Pro, 8 mm tip, Medtronic) was used in 531 patients, the POLARx catheter (POLARx 5 mm or 12 mm tip, Boston Scientific Corporation, Marlborough MA, USA) was applied in 65 patients (Table 1).

After trans-septal puncture, the balloon device was advanced to the LA via a steerable trans-septal sheath (15-F FlexCath advance Medtronic or 15.9-F POLARSTEATH, Boston Scientific).

**Figure 1:**
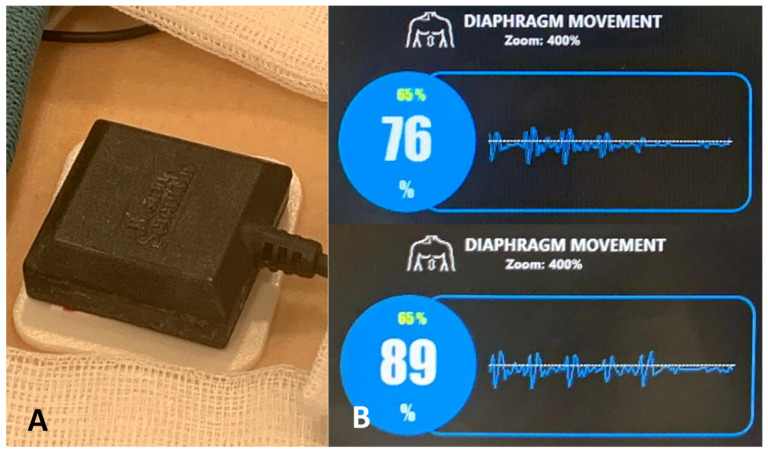
Novel diaphragm movement sensor (DMS). (**A**) The novel diaphragm movement sensor is placed on an electrode below the right-sided costal cartilage. (**B**) Section of the SMARTFREEZE Cryoablation Console surface with visualization of the DMS during pacing of the right-sided phrenic nerve and isolation of the right inferior pulmonary vein.

A multipolar mapping catheter (Achieve Advance Mapping Catheter, Medtronic or POLARMAP, Boston Scientific) was introduced for mapping of the PV potentials. A quadripolar catheter (Dynamic XT Boston Scientific or Inquiry Abbott) was used to confirm continuity of the phrenic nerve by pacing in the superior vena cava. Diaphragmatic excursion was assessed by continuous abdominal palpation and compound motor action potential (CMAP) visualization during ablation of the right sided PVs (RPVs) with the AFA catheter. When using the POLARx system, the novel diaphragm movement sensor (DMS) was applied (Figure 1).

**Figure 2:**
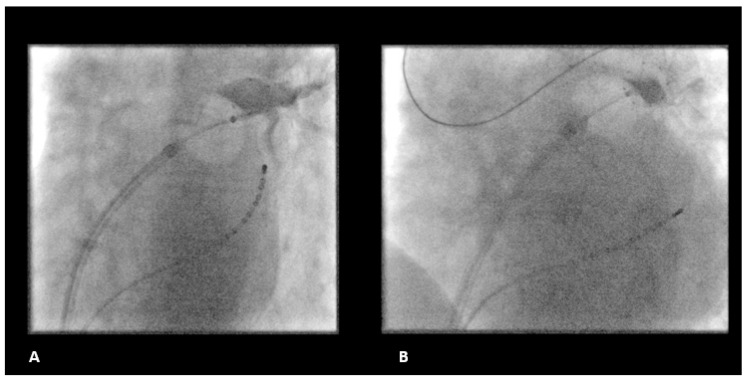
Fluoroscopic example of LSPV freezing. (**A**) LSPV freezing with the POLARx compared to the (**B**) AFA CBA catheter. CBA, cryoballoon ablation.

The degree of PV occlusion was evaluated by contrast injection after balloon inflation and placement and verified by repeat PV angiography in the initial freezing period (Figure 2). Ablation was performed adherent to a 2 × 180 s freeze per vein protocol. Persistent PVI (entrance and exit block) was confirmed after a waiting period of 20 min.

## 3. Statistical Analysis

All statistical analyses were performed with SPSS, version 24 (SPSS, Inc., Chicago, IL, USA). Continuous variables between the groups (POLARx and AFA) were compared by employing an unpaired two-sided Student’s t-test. Categorical data were examined by Pearson’s chi-square or two-sided Fisher’s exact test. Data are presented as mean ± standard deviation (SD) or percentage value unless stated otherwise. A *p*-value ≤ 0.05 was considered statistically significant.

## 4. Results

### 4.1. Baseline Characteristics

Baseline characteristics are summarized in Table 2. The POLARx group had a higher proportion of patients with hypertension (POLARx: 37 (57%) vs. AFA: 220 (41%), *p* > 0.001 *) when compared to the AFA group. Beyond that, significantly more POLARx patients suffered from PAF (POLARx: 43 (66%) vs. AFA: 281 (53%)) compared to AFA patients. Other baseline variables were similar between the groups.

### 4.2. Procedural Characteristics

POLARx group patients presented with a significantly longer procedure duration (POLARx: 113.3 ± 23.2 min vs. AFA: 100.9 ± 21.3 min, *p* < 0.001 *) and fluoroscopy time (POLARx: 10.5 ± 5.9 min vs. AFA: 4.8 ± 3.6 min, *p* > 0.001 *). Detailed procedural characteristics are summarized in Table 3.

### 4.3. Acute Procedural Outcome

Acute procedural success in terms of PVI was achieved in all patients (POLARx: 260/260 PVs (100%) vs. AFA 2112/2112: 100%, *p* = 1.000). Using the POLARx catheter significantly more PVs were isolated with the first freeze-application (POLARx: 192 (74%) vs. AFA: 1225 (58%), *p* = 0.027 *). Significantly more PVs were isolated within the 2nd freeze when applying the AFA catheter (POLARx: 64 (24%) vs. AFA: 96 (37%), *p* = 0.038 *). In both groups, it was rarely necessary to apply 3 freeze-cycles or more (POLARx: 4 (2%) vs. AFA: 92 (4%), *p* = 0.205). Overall, significantly more cycles per vein have been applied in the AFA group (POLARX: 1.3 ± 0.9 vs. AFA: 1.5 ± 0.7, *p* = 0.023 *). Acute success rates per individual freeze cycle are demonstrated in Table 4.

### 4.4. Cryoablation Freeze Temperature

The POLARx balloon achieved significantly lower temperatures during the freeze-application in all PVs. Detailed information is shown in Table 5.

### 4.5. Procedure-Related Complications

In the AFA group a periprocedural thromboembolic event was observed in one patient (<1%). Two patients suffered from PE with need for puncture (<1%), persistent phrenic nerve palsy (<1%) and vascular groin complications (<1%). In the POLARx group PE requiring treatment occurred in one patient (2%). In contrast to the AFA group no phrenic nerve palsy was documented.

In both groups, no esophageal perforation/fistula or death occurred. Details are presented in Table 6.

## 5. Discussion

This study has five major findings:

1. Both balloon-guided ablation systems are comparably safe and effective for acute single-shot PVI.
2. AF ablation utilizing the POLARx system is associated with longer procedure duration and fluoroscopy times. POLARx achieved higher isolation rates with the first freeze.
3. Nadir cryoballoon temperatures were significantly lower in the POLARx group.
4. No group differences were observed with regard to complication rates.
5. Long-term data and assessment of lesion formation are warranted.

### 5.1. Safety First

The novel POLARx cardiac CB-system was introduced to further facilitate balloon-based single shot PVI procedures due to its advanced design and features.

Approximately 4–14% of patients undergoing AF catheter ablation experience complications, 2–3% of which are potentially life-threatening [1]. Complications occur mostly within the first 24 h after the procedure [1]. Especially PEs appear to occur more frequently in the setting of RF-guided compared with CB-guided catheter ablations [22]. In our study complication rates were very low (Table 6) with comparable results in the AFA and the POLARx group. This acts in concert with previous studies reporting initial experiences with the POLARx catheter under clinical conditions with low complication rates for CB-guided PVI [17,19,20,21,23].

In contrast to one study reporting on a transient ST-elevation presumably caused by an air embolism possibly evoked by the larger sheath of the Boston cryoablation system (15.9 F) compared to the Medtronic system (15.0 F) [17] we did not observe any transient signs of myocardial infarction in our cohort of patients.

When using the POLARx system, the new DMS was applied. No phrenic nerve palsy occurred in the POLARx group (0%) compared to two documented persistent phrenic nerve palsy events in the AFA group (<1%) (Table 6). These comparable and low phrenic nerve palsy rates are in line with other studies [15,17,19,20,21]. Thus, further larger studies are certainly needed to elaborate potential benefits of the novel DMS.

### 5.2. Same but Different

As the POLARx catheter offers a stable size and equal balloon pressure during the inflation and ablation period unlike the AFA catheter, it might help to prevent from any kind of pop-out phenomenon as well as slight shifts of the balloon during the freezing cycle [17]. Thus, an exact coaxial alignment and only minimal push is required to achieve an adequate balloon-to-tissue contact. Beyond that, the handling of the POLARx system is comparably smart and straightforward due to improved material properties. Especially the POLARSTEATH appears to be softer and more flexible.

### 5.3. Things to Consider Using a New Ablation Device

Because of these innovations, a learning curve for the operator can certainly be assumed and may be one comprehensible reason for significantly longer procedure and fluoroscopy times using the novel POLARx system in the initial phase in our study (Table 3). These findings are in line with other studies [19,20,21]. However, some further studies have obtained only slightly worse or even similar results in terms of procedure and fluoroscopy times compared to those obtained under routine clinical conditions with the AFA [15,23]. A small study with 25 POLARx patients documented a trend towards even shorter procedure times compared to AFA treated patients [17]. Thus, data are still heterogenous. Beyond that it has to be taken into account, that in our study all procedures were performed by experienced electrophysiologists but not all examinations were conducted by the same operators. More experience needs to be gathered and worked up in larger studies.

### 5.4. Acute Procedural Success

In POLARx treated patients the grade of PV occlusion and the success at PVIs was comparable to the AFA group. All PVs could be reached and isolated (Table 4). Other studies have come to comparable conclusions [15,17,18,19,20,23].

In our study, significantly more PVs could already be isolated with the first freeze cycle using the POLARx instead of the AFA catheter (Table 4).

This could be due to improved features of the POLARx system offering optimized balloon-tissue contact for optimal effects of CB-guided catheter ablation.

However, other studies reported more freeze cycles [15,19,21,23], particularly in relation to the right pulmonary veins [15]. The authors of the latter study explain this by the lower stiffness of the multipolar mapping catheter as well as by excellent signals achieved with the POLARMap catheter [15]. Thus, more procedural experience and long-term results are needed in this context as well.

### 5.5. Minimal Freezing Temperature

All studies published to date, including ours (Table 5), agree that the POLARx catheter achieves significantly lower minimal freezing temperatures [15,17,18,19,20,23]. In summary, the optimal minimum temperature for the POLARx system seems to be about −5 to −10 °C lower compared to the AFA system [15,17,18,19,20,23].

This could be explained by differences in material properties, in expansion pressure or a slightly different position of the temperature probe within the POLARx system. Thus, dosing schedules do not seem to be identical to the AFA catheter. Further larger studies are needed to develop valid dosing regimens for the POLARx catheter resulting in reliable isolation of PVs.

## 6. Conclusions

This analysis demonstrated that both balloon-guided ablation systems are comparably safe and effective for acute single-shot PVI using cryoenergy. AF ablation utilizing the POLARx system is associated with longer procedure duration and fluoroscopy times as well as significantly lower nadir temperatures. Long-term data and assessment of lesion formation are warranted before further conclusions can be drawn.

## Figures and Tables

**Table 1:** Technical aspects of the POLARx compared to the AFA system.

Characteristics	POLARx	AFA
Sheath diameter (F)	12.7	12
Sheath outer diameter (F)	15.9	15
Radiopaque marker proximal to the tip (mm)	2.5	5
Balloon size (mm)	28	28
Balloon shaft diameter (F)		10.5
Balloon tip length (mm)	5 or 12	8
N_2_O injection	8-hole coil	8-hole coil
N_2_O fluid flow during freeze (sccm)	7800	7200
Pressure during freeze (psi)	<525 constant	530–600
Location of injection coil from pole of balloon (mm)	2.5	3.5
Location of TC from coil (mm)	18	15
Location of gas outflow proximal of TC (mm)	5	10
Phrenic nerve palsy control	DMS (integrated/quantitative)	CMAP (not integrated/not quantitative)
Console register procedural data	yes	no
Console operation autonomically	yes	no

CMAP, compound motor action; DMS, diaphragm movement sensor; TC, thermocouple.

**Table 2:** Baseline characteristics.

Characteristics	POLARx (n = 65)	AFA (n = 531)	*p*-Value
Age (years)	65.0 ± 11.6	63.0 ± 27.9	0.221
Gender, female	20 (31%)	132 (25%)	0.067
BMI (kg/m^2^)	30.6 ± 8.8	28.6 ± 5.7	0.060
LVEF (%)	52.8 ± 7.7	53.6 ± 4.1	0.137
Cardiomyopathy	8 (12%)	43 (8%)	0.087
Hypertension	37 (57%)	220 (41%)	**<0.001** *
Diabetes mellitus	7 (11%)	80 (15%)	0.104
Beta blocker	54 (83%)	421 (79%)	0.101
AADs	5 (8%)	40 (8%)	0.173
PAF	43 (66%)	281 (53%)	**0.018** *
Early recurrence	7 (11%)	51 (10%)	0.148

Continuous variables are shown as the mean ± SD and categorical variables as the number (%). A *p*-value ≤ 0.05 indicates statistical significance. BMI, body mass index, LVEF, left ventricular ejection fraction, LA, left atrium, AADs, antiarrhythmic agents, PAF, paroxysmal arterial fibrillation. * and bold letters indicate statistical significance.

**Table 3:** Procedural characteristics.

Characteristics	POLARx (n = 65)	AFA (n = 531)	*p*-Value
Total procedure time (min)	113.3 ± 23.2 [96.0, 130.0]	100.9 ± 21.3 [85.0, 114.0]	**<0.001** *
Total fluoroscopy time (min)	10.5 ± 5.9 [6.7, 12.5]	4.8 ± 3.6 [2.5, 6.2]	**<0.001** *
Contrast agent (mL)	38.1 ± 13.8 [30.0, 46.5]	42.9 ± 16.5 [30.0, 60.0]	0.075
Cumulative radiation dose (cGycm^2^)	432.8 ± 639.4 [116.0, 442.7]	519.9 ± 363.5 [242.0, 701.0]	0.300

Continuous variables are shown as the mean ± SD and as median (25th and 75th percentiles). A *p*-value ≤ 0.05 indicates statistical significance. * and bold letters indicate statistical significance.

**Table 4:** Acute success rates per freeze cycle.

	POLARx (n = 65)					AFA (n = 531)					*p*-Value
	LSPV (n = 65)	LIPV (n = 65)	LCV (n = 0)	RIPV (n = 65)	RSPV (n = 65)	LSPV (n = 519)	LIPV (n = 519)	LCV (n = 12)	RIPV (n = 531)	RSPV (n = 531)	
Isolation of PV (%)	100	100	-	100	100	100	100	100	100	100	1.000
Isolation with 1st freeze (%)	66	86	-	78	67	64	50	8	72	48	**0.027** *
Isolation with 2nd freeze (%)	32	14	-	20	30	33	48	42	23	46	**0.038** *
Isolation with 3rd freeze or more (%)	2	0	-	2	3	3	2	50	5	6	0.205

LSPV, left superior pulmonary vein; LIPV, left inferior pulmonary vein; LCV, left common vein; RIPV, right inferior pulmonary vein; RSPV, right superior pulmonary vein. * and bold letters indicate statistical significance.

**Table 5:** Cryoablation freeze temperature.

Characteristics	POLARx (n = 65)	AFA (n = 531)	*p*-Value
LSPV			
Minimal temperature (C°)	−58.2 ± 5.3 [−61.0, −55.0]	−46.0 ± 5.8 [−49.0, −43.0]	**<0.001** *
LIPV			
Minimal temperature (C°)	−56.9 ± 5.6 (−60.0, −53.0]	−41.3 ± 4.7 [−44.8, −39.0]	**<0.001** *
LCV			
Minimal temperature (C°)	N/A	−38.0 ± 14.2 [−43.0, −27.0]	N/A
RIPV			
Minimal temperature (C°)	−58.8 ± 6.5 [−63.0, −54.8]	−47.4 ± 7.1 [−52.0, −42.3]	**<0.001** *
RSPV			
Minimal temperature (C°)	−56.9 ± 7.6 [−62.0, −53.0]	−45.9 ± 6.7 [−51.0, −41.0]	**<0.001** *

Continuous variables are shown as the mean ± SD and as median (25th and 75th percentiles). LSPV, left superior pulmonary vein; LIPV, left inferior pulmonary vein; LCV, left common vein; RIPV, right inferior pulmonary vein; RSPV, right superior pulmonary vein. A *p*-value ≤ 0.05 indicates statistical significance. * and bold letters indicate statistical significance.

**Table 6:** Procedure related complications.

Characteristics	POLARx (n = 65)	AFA (n = 531)	*p*-Value
**Life threatening complications**			
esophageal perforation/fistula	0 (0%)	0 (0%)	N/A
Periprocedural thromboembolic event	0 (0%)	1 (<1%)	0.902
Cardiac tamponade	1 (2%)	2 (<1%)	0.065
**Severe complications**			
Persistent phrenic nerve palsy	0 (0%)	2 (<1%)	0.813
Vascular complications	0 (0%)	2 (<1%)	0.813
**Moderate or minor complications**			
Various	0 (0%)	0 (0%)	N/A

Ablation related complications classified according to the 2020 ESC Guidelines for the diagnosis and management of atrial fibrillation. Categorical variables are shown as the number (%). A *p*-value ≤ 0.05 indicates statistical significance.

## Data Availability

The data underlying this article will be shared upon reasonable request to the corresponding author.
